# Brucellosis: Review on the Recent Trends in Pathogenicity and Laboratory Diagnosis

**DOI:** 10.4103/0974-2727.72149

**Published:** 2010

**Authors:** Supriya Christopher, B L Umapathy, K L Ravikumar

**Affiliations:** Department of Microbiology, Kempegowda Institute of Medical Sciences, BSK II Stage, Bangalore, India

**Keywords:** Brucellosis, lipopolysaccharide, virulence, zoonotic disease, serodiagnosis

## Abstract

Brucellosis is a zoonotic infection transmitted from animals to humans by the ingestion of infected food products, direct contact with an infected animal or inhalation of aerosols. The last method is remarkably efficient given the relatively low concentration of organisms (10 – 100 bacteria) needed to establish infection in humans, and has brought renewed attention to this old disease. *Brucella* is a facultative intracellular pathogen that has the ability to survive and multiply in the phagocytes and cause abortion in cattle and undulant fever in humans. *Brucella* spp particularly *B. melitensis*, *B. abortus*, and *B. suis* represent a significant public health concern. At present, *B. melitensis* is the principle cause of human brucellosis in India. Molecular studies have demonstrated the phylogenetic affiliation of *Brucella* to Agrobacterium, Ochrobactrum, and Rhizobium. Human brucellosis still presents scientists and clinicians with several challenges, with regard to the understanding of its pathogenic mechanism, severity, progression, and development of improved treatment regimens. Molecular studies have now highlighted the pathogenesis of *Brucella*, for the development of newer diagnostic tools that will be useful in developing countries where brucellosis is a common, but often a neglected disease. This review compiles all these issues in general and the pathogenicity and newer diagnostic tools in particular.

## INTRODUCTION

From the time of the Roman era, organisms resembling *Brucella*e have been detected in carbonized cheese. Brucellosis was predominant in the Mediterranean region and its history is associated with military campaigns. This disease was fully elucidated by Sir David Bruce, Hughes, and Zammit working in Malta.[1] Bang discovered *B. abortus*, the causative agent of abortion in cattle and of brucellosis (undulant fever) in human beings.[2] The disease remains the world’s most common bacterial zoonosis, with over half a million new cases annually and the prevalence rate in some countries exceeds ten cases per 100,000 population,[3] being higher in people working in organized farms.[4] Despite being endemic in many developing countries, brucellosis is under-diagnosed and under-reported.[5]

Brucellosis in human beings is rarely fatal, but can lead to severe debilitation and disability. Nevertheless, it is reported that approximately 2% of the untreated patients die of brucellosis.[6] The disease has the tendency toward chronicity and persistence, becoming a granulomatous disease capable of affecting any organ system.[7] The timely and accurate diagnosis of human brucellosis continues to challenge clinicians because of its non-specific clinical features, slow growth rate in the blood culture, and the complexity of its serodiagnosis.[8,9]

Phylogenetically, *Brucella* is classified within the α 2 subdivisions of the Proteobacterium, which includes Agrobacterium, Rickettsia, Rhodobacterium, and Rhizobium.[10] Establishing a relationship within the genus has been challenging because of the relatively few genetic polymorphisms that distinguish each species.[11] Six species are recognized within the genus *Brucella*: *B. abortus*, *B. melitensis*, *B. suis*, *B. ovis*, *B. canis*, and *B. neotomae*. This classification is based on the differences in pathogenicity and host preference.[12]

In recent times, two new species have been added to this genus, *B. cetaceae* and *B. pinnipediae*, isolated from marine mammals, cetaceans, and pinnipeds.[13] The *Brucella* genome consists of two circular chromosomes, without plasmids, suggesting a remarkable difference compared to the single chromosome of many bacteria.

Successful infection by pathogenic bacteria often depends on their ability to survive and multiply within the host cells. To do so, they alter or adapt to the host cell environment. To these ends, pathogenic bacteria contain a variety of secretion systems, including type I, II, III & IV systems which can export virulence factors to the environment or into the infected host cell.[14] However some of the *Brucella spp* lack these secretion system, except for some like *B. melitensis* contains genes for flagellum- specific type III and IV secretion systems.[15] These secretion systems are involved in variety of process ranging from the delivery of virulence factors into the eukaryotic cell to conjugation, transfer of genetic material, uptake or release of DNA.[16] The recent completion of *B. melitensis* (Gene Bank NC003317) and (NC003318),[17] *B. suis* (Gene Bank NC002969), and the *B. abortus*[18] genome sequence projects have provided tremendous information for understanding the mechanisms of *Brucella* pathogenicity. The availability of the complete genome sequences and advancement of genomics and proteomics has enabled scientists to understand the disease and its pathogenic mechanisms. The development in culture and serological methods are routinely used for the diagnosis of the disease, however, advanced molecular detection and typing methods have contributed to improving the laboratory diagnosis. This article reviews and summarizes the current knowledge of the pathogenic mechanisms and the newer diagnostic advances made in human brucellosis.

## PATHOGENICITY

*Brucella* spp are facultative intracellular bacteria that have the ability to avoid the killing mechanism and proliferate within the macrophages, similar to other intracellular pathogens.

To be a successful infectious agent, *Brucella* requires four steps: adherence, invasion, establishment, and dissemination within the host

Opsonised and non opsonised *Brucella* can infect macrophages. Thereby indicating direct host cell contact which allows adherence and invasion as well as antibody or complement mediated phagocytises. In the macrophages. *Brucella* cells survive and multiply, inhibiting phagosome–lysososme fusion. Finally, the accumulated bacteria are disseminated to other host cells.[15]

After infecting the host, the pathogen becomes sequestered within the cells of the reticuloendothelial system. The mechanism by which *Brucella* enters the cells and evades intracellular killing and the host immune system is a subject of much research and debate.

Several studies on the virulence factors are directed at the main components of the outer membrane. The outer membrane contains Lipopolysaccharide (LPS), which is the major virulence factor of *Brucella*. It possesses a peculiar non-classical LPS as compared to the classical LPS from Enterobacteria, such as *Escherichia. coli*[19] [Table 1].

**Table 1:** Difference between Classical & Non-classical LPS

Classical LPS	Non classical LPS
Exhibit high toxicity	Exhibit low toxicity for endotoxin sensitive mice and rabbit
High pyrogenicity	Low pyrogenicity
Inducers of interferons and tumor necrosis factor	Weak inducers of interferons and tumour necrosis factor
Examples: *E. coli*	Example: *Brucella. abortus*

Smooth LPS has a role in cell entry and immune evasion of the infected cell. It also alters the capacity of the infected cell to present foreign antigens to the MHC class II antigen presentation system, hence, preventing the attack and killing the infected cell with the help of the immune system.[20] LPS has three domains:

Lipid A, the core oligosaccharide, and the O-antigen.

The O-polysaccharide of the smooth type *Brucella* LPS is an unbranched homopolymer of 1-2 linked 4, 6 dideoxy-4-formamido and α-D mannopyranosyl, usually with an average chain length of 96 to100 glycosyl subunits.[21]

The O-polysaccharide is linked to the core polysaccharide composed of mannose, glucose, 2–amino-2, 6–dideoxy–D-glucose, 2–amino–2–deoxy–D-glucose, 3 deoxy–D–manno–2–octulosonic acid (KDO), and unidentified sugars. (The lipid A linked to the core polysaccharide contains 2, 3-diamino-2,3 dideoxy-D-glucose as the backbone and amide- and ester-linked long chain saturated (C _16:0_ to C _18:0_) and hydroxylated fatty acids.[22]

The heterogeneity of the enterobacteria is known to be related to the length of its O-polysaccharide and different chemical substitutions in the core oligosaccharide and lipid-A.[23] In the enterobacterial lipid A, the degree of heterogeneity depends on the different combinations in which the amide- and ester-linked fatty acid, phosphates, neutral sugars, ethanolamine, and different types of backbone amino sugars occur in the molecule,[24] whereas, in *Brucella* lipid A, the degree of heterogeneity depends mainly on various fatty acid substitutions. There is an absence of backbone constituents and ester-linked acyl-oxyacyl residues in *Brucella* lipid A, as compared to enterobacterial lipid A.[25] Determination of heterogeneity in *Brucella* LPS is important for practical purposes, as it is the most relevant antigen during infection and vaccination. The genome sequences of *B. melitensis, B. suis*, and *B. abortus* have become available recently.[26] They are similar in sequence, organization, and structure. Comparative genomics provides an insight into the aspects of *Brucella* virulence that has only been suspected earlier. Using the complete genome sequence of *B. melitensis*, Dricot *et al*.[27] has generated a database of protein coding ORFs and constructed an ORFeome library of 3091 gateway entry clones, each containing a defined ORF. The genome sequence of the *B. melitensis* strain 16M contains 3,294,935 bp, which is distributed over two circular chromosomes of 2,227,144 bp and 77,787 bp, encoding 3,197 ORFs. The genome of *B. abortus* biovar (strain 9-941) has 3.3 Mb composed of two circular chromosomes of 2,124,242 (Chr I) and 1,162,780 bp (Chr II).[28] The genome of *B. suis* is a 1330 genome consisting of two circular chromosomes of 2,107,7892 bp and 1,207,381 bp.[29]

The genome of *B. abortus* shares more fragments with *B. suis* and *B. melitensis*, than *B. suis* and *B. melitensis* do with each other. A majority of the genes studied are involved in *Brucella* biosynthesis and O–chain synthesis-like persominesynthetase (per), mannosyl tranferase (wbkA, WbdA, B, C), phosphoglucomutase (pgm), ABC type transporters (W_2m,_W_2t_), and mannose (manA,B,C).The BvrR / BvrS gene sensing system acts as a cascade of protein phoshorylation to modulate the key expression, and these key factors are involved in cell binding and penetration. This system has an effect on the expression of cell surface proteins Omp 25 (Omp3a) and Omp 229 (Omp3b).[30]

This altered cell expression of the surface proteins allows *Brucella* to bind to and penetrate the lysosomal pathway. A type IV secretion system (Vir B) selectively transports proteins and macromolecules through the membranes and is essential for intracellular survival, in case of *Brucella*. It also helps in adherence of the bacterium to the host cell and cell entry.[31] A large number of attenuated mutants, with structural defects in their Lipopolysaccharide, confirm the importance of this molecule in *Brucella* virulence.[32] Heat shock protein 60 (Hsp60), a member of the GroEl family of chaperonins, is expressed on the cell surface of wild-type *Brucella* spp, but not on VirB mutants. Hsp60 seems to play a part in cell adherence by binding to a cellular prion molecule called PrPr. As the exportation of Hsp60 is VirB-dependent, it has been postulated that Hsp60 may in fact be a virulence factor.[33] Once the organism binds to the macrophages, the internalization vesicles that would fuse with the endosomes take it up. The endosomes are lysed by acidification. This acidification is thought to induce Vir B expression.[34]

## DIAGNOSIS

Diagnostic methods for brucellosis are primarily based on serology, with the LPS smooth chains producing the greatest immunological responses in various hosts. The major diagnostic problem is due to the similarity of the O-antigenic side chain of LPS of *Brucella* and other organisms like *Yersinia enterocolitica* O : 9, *Vibrio. cholerae, Esherichia. coli* 0 : 157, and *Francisella. tularensis*. Alternative antigens have been evaluated for their diagnostic potential, for a possible improvement in its specificity, however, these have largely been unsuccessful. (Blood culture is the gold standard in the diagnosis of bacterial infections including brucellosis, but this method is successful in only 40 – 70% of the cases. The Biphasic Ruiz-Castaneda system is the traditional method for the isolation of *Brucella* sps in clinical samples.[35] It has been largely replaced by the lysis centrifugation technique, where a higher rate of positive blood culture has been reported. An automated culture system has also improved the speed of detection.[36] Bone marrow cultures may provide higher sensitivity, yield faster culture times, and may also be superior to blood culture, when evaluating patients with previous antibiotic use. *Brucella* can also be cultured from pus, tissue, cerebrospinal fluid (CSF), and pleural / joint / ascitic fluid.[37]

However, the results have not yet been universally reproducible.

## SERODIAGNOSIS

In the absence of culture facilitates the diagnosis of brucellosis relies on agglutination tests, such as, the Rose Bengal test, serum agglutination test, the antiglobulin or Coombs test, complement fixation test, and the recently introduced immunocapture test.

The Rose Bengal test is used as a screening test and positive results are confirmed by the serum agglutination tests.[38] This agglutination test is based on the reactivity of antibodies against the smooth lipopolysaccharide. In the Rose Bengal Plate (RBPT) agglutination test the sensitivity is high (>99%) and false negative results are rarely observed. To increase the specificity the test may be applied to a serial dilution (1:2 through 1:64) of the serum samples.[39] The Standard Tube Agglutination Test (SAT) developed by Wright and colleagues remains the most popular and easy test to perform. SAT can measure the total quantity of the agglutinating antibodies (IgG and IgM). The quantity of specific IgG is determined by treatment of the serum with 0.005M 2 mercaptoethanol (2ME), which inactivates the agglutinability of the IgM. However, many patients have low levels of agglutinating IgG antibodies and the results can easily be misinterpreted. SAT titers above 1 : 160 are considered diagnostic in conjunction with a compatible clinical presentation, however, in endemic areas the titer of 1 : 320 is taken as the cut off. Coomb’s test is the most suitable and sensitive test for confirmation in relapsing patients with persisting disease, but it is complex and demands technique. Enzyme linked immunosorbant assay (ELISA) has become increasingly popular, as well as a standardized assay for brucellosis. It measures IgG, IgM, and IgA, which allows a better interpretation of the clinical situation. The specificity of ELISA, however, seems to be less than the agglutination tests. As the diagnosis of *Brucella* is based on the detection of antibodies against smooth LPS, the cut-off value needs to be adjusted, to optimize the specificity when used in endemic areas.[40] ELISA can also be applied in the diagnosis of CNS brucellosis with varying success and further research must be aimed at improving the diagnosis of this condition. The Fluorescence polarization assay (FPA) offers a valuable alternative to conventional serological tests. This assay measures the size of a florescent tagged molecule such as an antigen — ideally antigens selected for this technique should be small (20 Kda). The utilization of the O-side chain of LPS from *Brucella* spp has shown encouraging results.[41] The sensitivity of this test at the selected cut-off value is 96% for culture-confirmed brucellosis and the specificity is 98%.[42]

Immunochromatographic *Brucella* IgM / IgG lateral flow assay (LFA), a simplified version of ELISA has a great potential as a rapid point-of-care assay. Studies have shown that this test has high sensitivity and specificity for *Brucella* IgM and IgG. This system uses a drop of blood obtained by a finger prick, which is used by the bedside and easy to interpret. It is a rapid and simple diagnostic test for confirmation of brucellosis in an endemic area.[43,44] In recent years new immunocapture agglutination for anti-*Brucella* (*Brucella* Capt BCAP) has been developed, to detect agglutinating and non-agglutinating antibodies with high sensitivity. It has been suggested as a possible substitute for Coombs test and a better marker for disease activity.[45]

## MOLECULAR DETECTION

Over the past decade there has been a major advancement in all aspects of molecular diagnostics with regard to human brucellosis. Polymerase chain reaction (PCR)-based tests are proving to be faster and more sensitive than the traditional methods.

Several genus-specific PCR systems using primer pairs that target 16SRNA sequences and genes of different outer membrane proteins have been developed (Queipo-Ortuno and co-workers found 100% sensitivity and 98.3% specificity by using a B4 / B5 primer and amplifying a 223-bp fragment of the *bcsp31* gene compared with 70% constituents of blood culture.[46]

Incorporation of a robust DNA extraction method, such as the diatom-guanidinium isothiocyanate method, effectively removes the inhibitors commonly present in a variety of clinical specimens and may improve the sensitivity and reproducibility.[47] However, as these PCR systems carry a high risk of contamination and require equipments for visualization, they are less suitable for routine diagnosis purposes. Hence, real time PCR systems have been developed that are faster and less prone to contamination and are thus more clinically useful.[48]

Relapsing brucellosis is another diagnosis challenge where PCR may prove to be useful. Nowadays, this is also used to assess treatment efficacy.[49] PCR is also useful in species differentiation and biotyping of isolates. There are some short nucleotide repeat sequences that are present in the *Brucella* genome showing a wide variation in the number of repeats between species and isolates. PCR amplification of these variable repeats is more robust than the classic typing methods for species and biovar identification.

This application could be applied epidemiologically to trace infections to specific flocks or dairy producers. One of the main characteristics of brucellosis is its marker tendency to relapse after completion of the treatment. This problem results from the intracellular location of the *Brucella* spp, which protects the bacteria from some of the basic mechanisms of the immune system, as well as from therapy. Relapses most frequently occur within six months to as long as two years of the initial treatment. Hence, it is necessary to monitor the patients during 12 months of the treatment.[50]

Morta and coworkers recently evaluated the usefulness of a PCR-based assay in a post-treatment follow up and relapse of patients with brucellosis.[51] There are several PCR assays for the detection of *Brucella* DNA using pure culture, animal, and human clinical samples. However, the sensitivity and specificity of PCR for *Brucella* varies between laboratories, and hence standardization is required.[52]

## CONCLUSION

Interest shown by the scientific community toward brucellosis has benefited developing countries like India. The DNA–DNA hybridization studies revealed a high degree of homology shared by six recognized species of *Brucella*. The genomic rearrangements, species-specific DNA sequence, and distinct patterns of gene inactivation suggest that *B. abortus* and *B. melitensis* share the same lineage, which differs from the *B. suis* lineage, which has undergone fewer genetic mutations, as it diverged from the most recent common ancestor of all *Brucella*. Although there are newer insights to the pathogenesis of *Brucella* spp, a lot of development is needed in the aspect of treatment, as *Brucella* spp do not follow the classical method of virulence, which has made investigation in this area slower. As brucellosis poses health threats to humans, and morbidity in untreated diseases is substantial, thus early consideration and diagnosis of brucellosis is important. As brucellosis is often misdiagnosed or overlooked, physicians in both endemic and nonendemic areas must be aware in their diagnosis of febrile diseases.
